# Russian Flu in Iran from 1889 to 1894

**DOI:** 10.34172/aim.33524

**Published:** 2025-04-01

**Authors:** Seyyed Alireza Golshani, Ghobad Mansourbakht, Ghazaleh Mosleh

**Affiliations:** ^1^Department of History, Shahid Beheshti University, Tehran, Iran; ^2^Department of Phytopharmaceuticals, School of Pharmacy, Shiraz University of Medical Sciences, Shiraz, Iran

**Keywords:** Grippe, Iran, Pandemic, Russian flu, Tabriz, Tehran

## Abstract

The ‘Russian flu,’ also referred to as the ‘Asiatic flu,’ spread globally between 1889 and 1894. According to estimates from international organizations, this epidemic resulted in the deaths of approximately one million individuals. However, there is no information available on the exact number of deaths in Iran. The earliest outbreak of the epidemic was reported in May 1889 in Bukhara, Central Asia, which was part of the Russian Empire. The Russian Railway facilitated the spread of the epidemic from Siberia to the easternmost regions of Russia, westward to Moscow, and subsequently to countries such as China, Sweden, Finland, and Western Europe, eventually reaching the United States and Argentina. It subsequently spread from southern Russia to the South Caucasus and Baku, then moved into Iran from the north, northeast, and northwest, suddenly appearing in cities such as Bandar Anzali, Sari, Rasht, Mashhad, Tabriz, Tehran, Isfahan, Shiraz, and Kerman. The epidemic caused unexpected casualties in the country and startled both modern and traditional physicians. Notably, this epidemic, which appeared in Iran in two waves during 1890 and 1892, was somewhat mitigated due to the country’s insufficient transportation infrastructure. As Tehran and Tabriz were either overpopulated or closer to Russia, doctors in these cities witnessed more cases of the Russian flu, prompting them to write several medical dissertations on this epidemic. This study examines the Russian flu in Iran as documented in historical, journalistic, and medical records.

## Introduction

 One of the points that receives less attention in the field of medical history is the geographical origin and linguistics of each disease. Many historians mistakenly believe that the outbreak of the flu was firstly reported in Italy, and that the word influenza in Italian means “heavenly disaster.” However, the disease, commonly referred to as “flu,” has an interesting etymology, derived from the two Arabic words “goat’s nose” [*Anf-ol-Anzah*= أنفُ العَنزة]. Iranian pioneer physicians such as Rhazes (865–925 CE) and Avicenna (980 –1037 CE) coined this term because patients’ sneezing resembled a goat’s sneeze, or their noses appeared goat-like. This disease is also known as “rhinitis” and “coryza” [نزله و زکام].^1-4^ Later, their books were translated into Latin, coining the term ‘influenza,’ which led to Italy and Europe being associated with its origin.^5^

 The most massive influenza epidemics of the nineteenth-century, known as the “Russian Epidemic” or “Asiatic flu” or “Russian flu,” reached Europe from the east in 1889.^6^ The first report of the disease was in May 1889 in Bukhara, Central Asia. It spread via the Trans-Caspian railway to cities like Samarkand and Tomsk. The incomplete Trans-Siberian railway slowed its spread to the east, but it eventually reached Türkmenbaşy and spread through the Volga trade routes. By November, it had reached Saint Petersburg, infecting 180 000 people in a city with about one million residents, and also spread to Moscow.^7,8^

**Figure 1:**
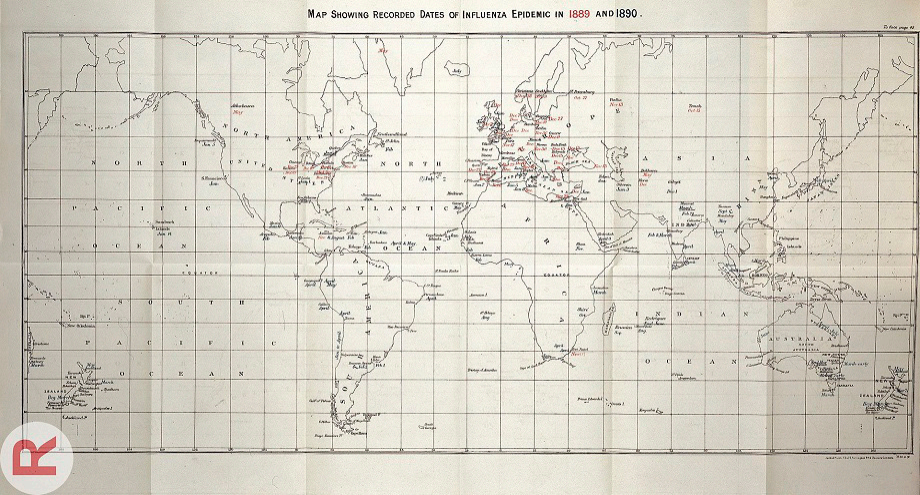
Map, Report on the Influenza Epidemic of 1889–91. Parsons, Henry Franklin (1891). Local Government Board^9^

**Figure 2:**
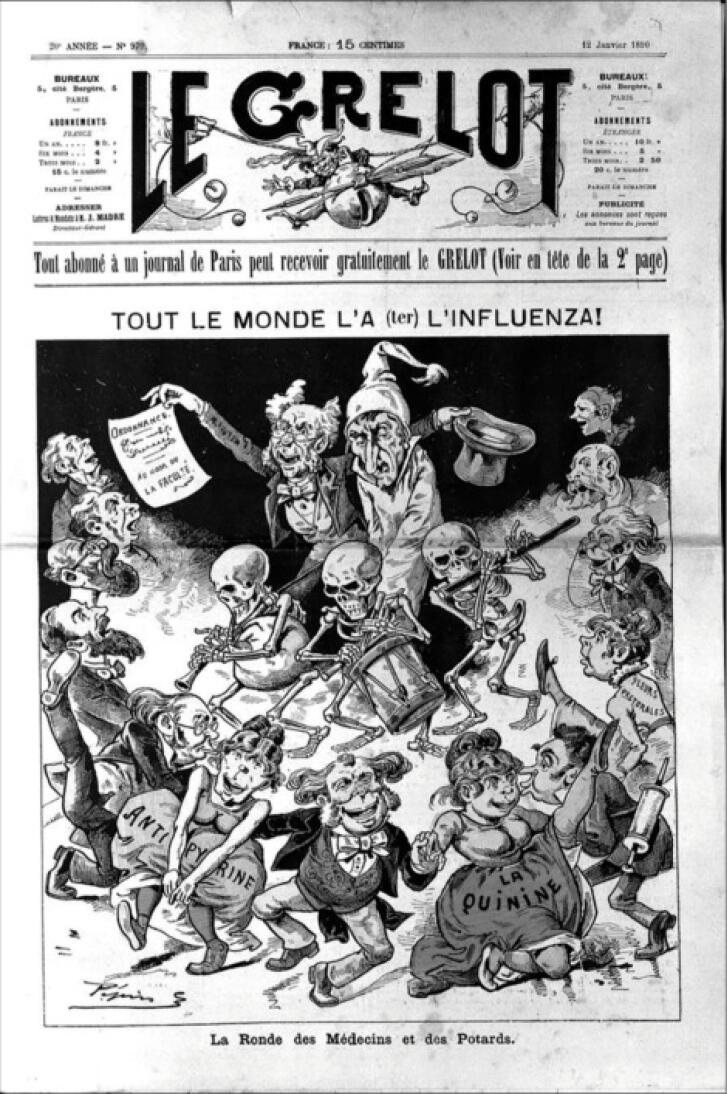
A Man with Influenza, Taken in Hand by a Doctor, Surrounded by Dancing Politicians. Wood Engraving by Pépin (E. Guillaumin), January 12, 1890. Wellcome Collection^10^

 By mid-November, Kiev was infected, followed by Lake Baikal in the subsequent month. By the year’s end, Siberia and Sakhalin were affected. The Nordic region was contaminated via the Baltic trade from St. Petersburg to Vaxholm in early November 1889, quickly spreading to Stockholm and infecting 60% of Sweden’s population within eight weeks. Norway and Denmark were soon infected. The German Empire saw its first case in December in Posen, and by November 12, 600 workers in Berlin and Spandau were infected, with half of Berlin’s residents affected within days.^7,8^ By December 17, Vienna and Rome were infected. The flu spread to Paris, other parts of France, and Spain, with a high mortality rate in Madrid. It quickly spread throughout Great Britain and Ireland. The first American flu case was reported on December 18, 1889, and within days, it spread along the East Coast to Chicago, Kansas, and San Francisco, causing 13 000 deaths. The epidemic then moved south to Mexico and Buenos Aires by February 2^7,8^ (Figures 1 and 2).

 The literature on the Russian flu in Iran is limited. Most studies briefly mention the flu’s spread between 1890 and 1893 without detailed information on the epidemic or affected regions. Floor, Afkhami, Alipoor Silab et al, and Vaziniafzal and Moosavi discuss the epidemic, noting its origin in the Russian Empire and its spread to Iran.^11-14^

## Influenza in Iran

 Iran has experienced the spread of four influenza virus types (A, B, C, and D). Dr. Willem Marius Floor, a historian and Iranologist, documented multiple influenza outbreaks in the Middle East, including in 855 AD in Iran, 1654 in Baghdad, 1753 in Basra, the winter of 1833 in Iran, Egypt, Syria, and Turkey, and October 1854 in Iran.^11,15,16^ During the winters of 1877 and 1878, a horse flu epidemic known as *Mashmasheh* caused the deaths of 2%-3% of foreigners in Iran, with unknown Iranian casualties. According to Dr. Willem Marius Floor, flu epidemics also occurred in Iran in 1890, 1891, 1895, 1897, 1910, 1912, and 1913, causing deaths but not as significantly as the 1918 epidemic.^11,16^ In the summer of 1833, the earliest verified evidence of the Russian flu in Iran emerged, spreading extensively in Tehran through commerce lines between Damascus and Istanbul (In the territory of the Ottoman Empire). This international epidemic affected thousands in Asia and Europe, causing numerous daily deaths in Tehran, with corpses found on the streets.^17^ Cyril Lloyd Elgood M.D. (1893-1970) noted that even the king, Fath-Ali Shah Qajar, was infected, experiencing severe fever and pain, and dozens of people died every day.^18^

## Russian Flu in Iran

 An unusual respiratory disease, called “Russian flu” or “Asiatic flu,” spread from Bukhara between 1889 and 1894, affecting the Russian Empire’s Transoxiana region and eventually the world. This epidemic, which peaked at least three times, is speculated by some scientists to be an early version of SARS-CoV-2, the virus responsible for COVID-19.^19^

 The Dutch historian Willem Floor describes the Russian flu without naming the disease: “On January 3, 1890, a flu spread in Tehran, and a more severe variant was seen in Tabriz.” It also emerged in other cities including Sari, Babol, Rasht, Isfahan, Mashhad, Shiraz, and Bushehr. In 1891, the flu became a severe disaster, killing many people on Bahrain Island, Qeshm Island and Qatif (an urban area in Saudi Arabia). From April to October 1893, the epidemic affected Bandar Gavater on the coast of Sistan and Baluchestan Province, as well as North Karun. It killed numerous people in Bandar-e-Jask (Hormozgan province) every day.^11^

 An examination of magazines and newspapers from the reign of Naser al-Din Shah Qajar (1848-1896) reveals terms such as Egyptian coryza, coryza fever, and odd [*Gharib*: غریب] disease describing the Russian flu. This disease appears to have caused more fatalities than any other, except cholera. Newspapers from the era provide comprehensive and detailed accounts of the epidemic’s spread in Europe and the United States.^20^ Magazines and newspapers, including Akhtar, reported that the epidemic originated in Russia and spread globally. It reached Iran through three main routes: from the Russian Empire and Bukhara, from the Arabian Peninsula and Egypt to the Oman Sea and Persian Gulf, and through Tabriz via the Balkan Peninsula and Istanbul, affecting populations in these areas.^20,21^

 Meanwhile, Joseph Désiré Tholozan (1820–1897), Naser al-Din Shah Qajar’s physician and a French epidemiologist, detailed how the Russian flu arrived in Tehran: “When in 1889 the Russian flu was spread into the western world through Russia, it took all of western Europe, and in September of the same year it came to Iran through Rasht in North Iran, and finally it reached Tehran”.^12^ The flu spread rapidly to Tabriz and by mid-November 1889, it reached Tehran again. On March 14, 1890, the epidemic arrived in Bushehr via the Persian Gulf, infecting at least half of Iran’s population. Initially mistaken for a common cold, the flu’s severity shocked the public and young doctors trained in Western methods. In Tehran, it caused 50 to 70 deaths daily, including prominent individuals like Mirza Kazem Khan Nizam al-Molk, head of the Ministry of Armed Forces.^12-22^

**Figure 3:**
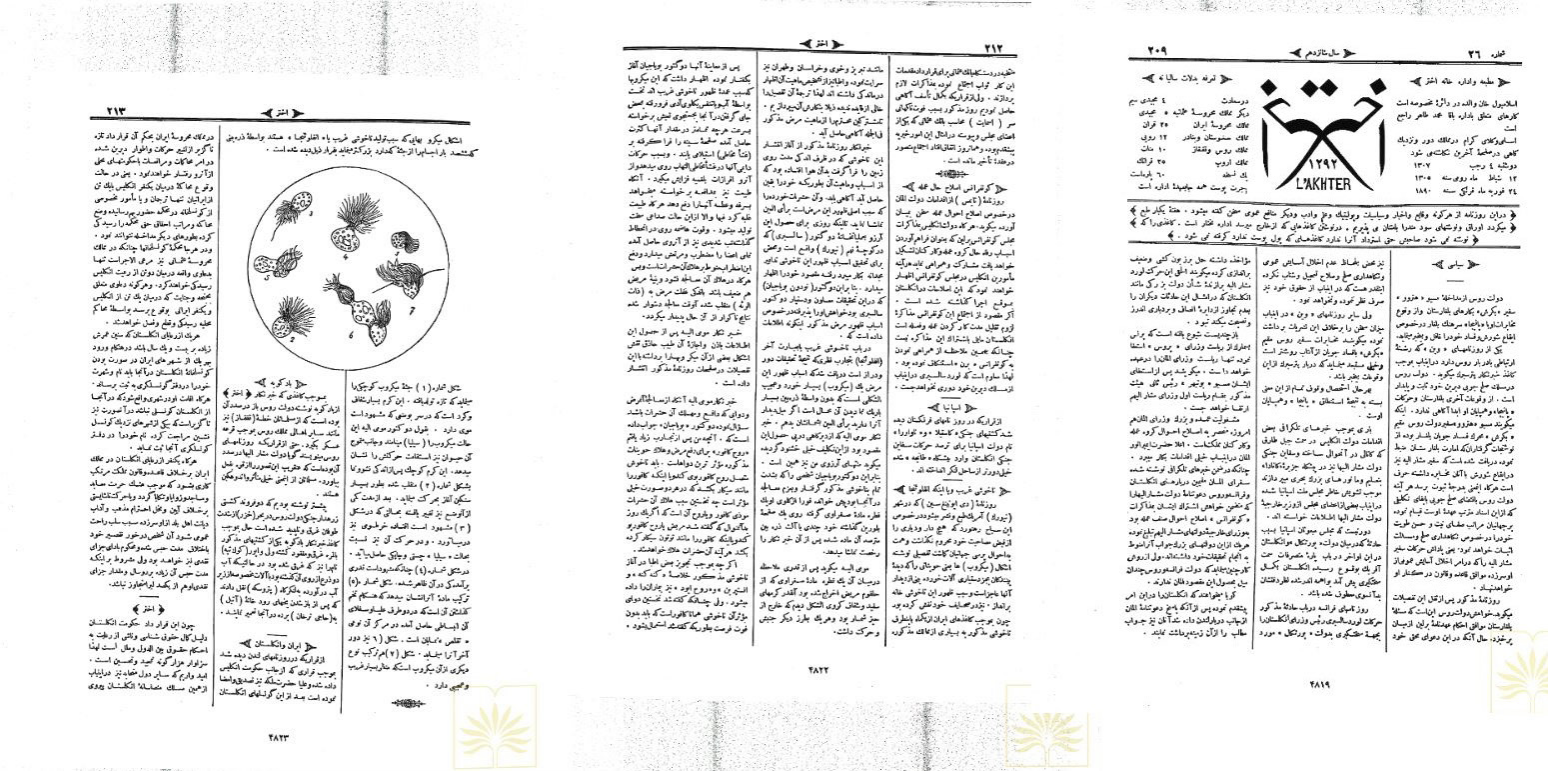
Akhtar Magazine, February 24^th^, 1890^24^

 The prevalence of the Russian flu was notably high among children, resulting in the deaths of over six thousand kids due to associated diseases like severe sore throat and measles. Iranian physicians, unaware of the illness’s nature, advised people to keep their homes warm and avoid cold exposure. They also prescribed detoxification medicines to clear toxins from the body and used quinine and camphor to alleviate fever and chills.^23^ The spread of the Russian flu in Iran led to the incorporation of the word ‘flu/influenza’ into the Persian language, which was previously absent until 1889. This occurred when the Istanbul-published Akhtar mentioned the flu and even included a schematic figure of the microbe^14,24^ (Figure 3).

 The publication accused Iranian physicians of exacerbating the epidemic’s death rates by misidentifying the flu as the common cold and failing to take immediate action. Consequently, the term “flu,” adopted from Europe, replaced the incorrect label “cholera” for this epidemic. The 1889 epidemic significantly introduced the idea to the educated class that microbes cause diseases. The peculiar and devastating nature of the flu heightened public interest in microbes and microorganisms, promoting advances in diagnosing and treating the disease.^12,24^ (Akhtar, February 24th). Abdulla Mustovfi (1877–1950), an Iranian writer, described the disease as a severe coryza or pneumonia that even killed his father. He states in a sarcastic tone, “These newcomer-educated doctors of Dār ul-Funun (meaning: polytechnic college) use the new word *flu* to speak of the disease”.^25^

 The flu epidemic that swept through Iran from 1889 persisted until the spring of 1890. With the arrival of summer, the disease began to gradually decline.^12^

**Figure 4:**
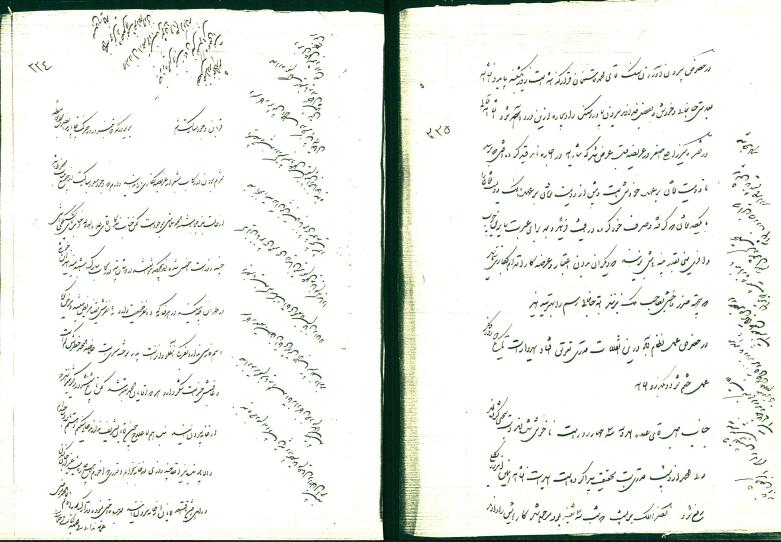
A Report on the Severe 1892 Influenza Outbreak in Tehran, Including the Death of Qajar Politician Mirza Nasrullah Khan Nasr al-Mulk (1827-1892) from the Russian flu^27^

 In 1892, during the reign of Naser al-Din Shah Qajar, the epidemic resurfaced in Tehran and spread rapidly throughout Iran. Jean-Baptiste Feuvrier, a French doctor and Naser al-Din Shah’s physician, documented the resurgence in his diaries: “The outbreak of the flu coincided with a mild winter. Since the end of November, it has only been cold for a few days and snowed once; the sky has been clear and the temperature has not been unbearable, except for last night’s severe storm, which turned the blue sky gloomy. What a severe storm...!”.^26^ Feuvrier’s account of the recurrence in Tehran on January 23, 1892, is as follows: “The flu, which has been emerging for days in all Tehran neighborhoods, has claimed the lives of many people. Mirza Yahya Khan Moshir od-Dowleh, an Iranian minister and statesman, was among the first to die from the disease three days ago (January 20th, 1892). Also, today, two female residents of the palace died. Given this, the king shouldn’t visit the city. These days, we were discussing my return. But the news about the disease resurgence caused this decision to be delayed”.^26^ Feuvrier documented in his January 28th report that the journey between Tehran, Tappeh, and Dowshan ultimately brought the flu to Tappeh and Dowshan, where the Shah resided: “This morning, Shah, who had spent the night in fever and unrest, woke up while feeling coming down with a cold.” Feuvrier’s report, dated February 7^th^, states that Shah recovered from the flu^26^ (Figure 4).

 Mashhad, Kerman, and Shiraz were among the cities affected by the epidemic, although detailed reports are lacking. It was noted that Mashhad experienced the flu in 1890, resulting in several fatalities. Local history books indicate that Kerman faced its first Russian flu epidemic in 1891, likely spreading from southern Iran and Persian Gulf harbors to Kerman and Shiraz.^28-30^ On November 28, 1893, the Russian flu emerged and circulated in Shiraz, affecting approximately one-third of the population and resulting in several deaths.^13^

## Russian Flu and Iranian Physicians

 Like other parts of the world, the Russian flu reached Iran, with Tabriz being one of the first affected cities. The emergence of influenza in 1890-1891 prompted the Philosopher al-Dawla Mirza Abdolhossein Zonuzi Tabrizi (1866-1941) to create “Grippe” or “treatise on influenza” in 1891, nearly twenty-seven years before the Spanish flu of 1918.^31^ The compilation of the “very important influenza” or “treatise on flu” is one of the first compilations in contemporary history by an Iranian physician influenced by European medical treatises.^32^ The experience of dealing with the “Russian influenza” in Tabriz, the enhanced expertise of physicians, and the insights from this dissertation might be crucial factors contributing to the relatively low mortality rate during the 1918 flu outbreak in Tabriz and Azerbaijan.

## Philosopher al-Dawla Mirza AbdolhosseinZonuzi Tabrizi

 “Mirza Abed al-Hussein Khan” was born into a cultural and scholarly family in 1886 in Tabriz. He went on to become one of the leading physicians in Azerbaijan, located in northwestern Iran.^31,32^ He learned the basics of Iranian medical sciences in Tabriz, then studied in Tehran under Joseph Désiré Tholozan (1820-1897), a French physician specializing during the reign of Naser al-Din Shah Qajar (1831-1896). After returning to Tabriz from Tehran, equipped with sufficient knowledge, he dedicated his time to advancing science and medicine in Azerbaijan, working under the supervision of Seyyed Ali Sayed al-Hikma and other renowned physicians.^13,14^ Zonuzi made substantial contributions to both scientific and practical medicine in Tabriz over many years, authoring several notable books. He played a pivotal role in advancing modern medicine in Tabriz and Azerbaijan, effectively integrating traditional medical knowledge with contemporary scientific advancements and educating the public on modern medical practices. Additionally, he served as the physician for Mohammad Ali Shah Qajar, the Qajar Crown Prince (1872–1925) during his residency in Tabriz.^32,33^

 One of Zonuzi’s distinguished works, “Excerpted from the Tragedy of the Al-’Asar,” is a biographical encyclopedia featuring the biographies of 406 medical and philosophical figures. This meticulously written work has gained significant attention and has been extensively cited in medical history texts over the past 50 years. Among his other notable contributions are “History of Tabriz,” “Al-Jadariyah,” “Leprosy,” “Footnotes on Euclid’s Interpretations,” “Marfat al-Somom Nasseri” (Toxicology Knowledge), and “Muftah al-Adawiyah” (The Key to the Drugs).^13,32,33^

 He lived in Tabriz for many years before moving to Qom, where he headed the hospital. Later, he went to Mashhad, where he eventually passed away in 1941.^31,32^

**Figure 5:**
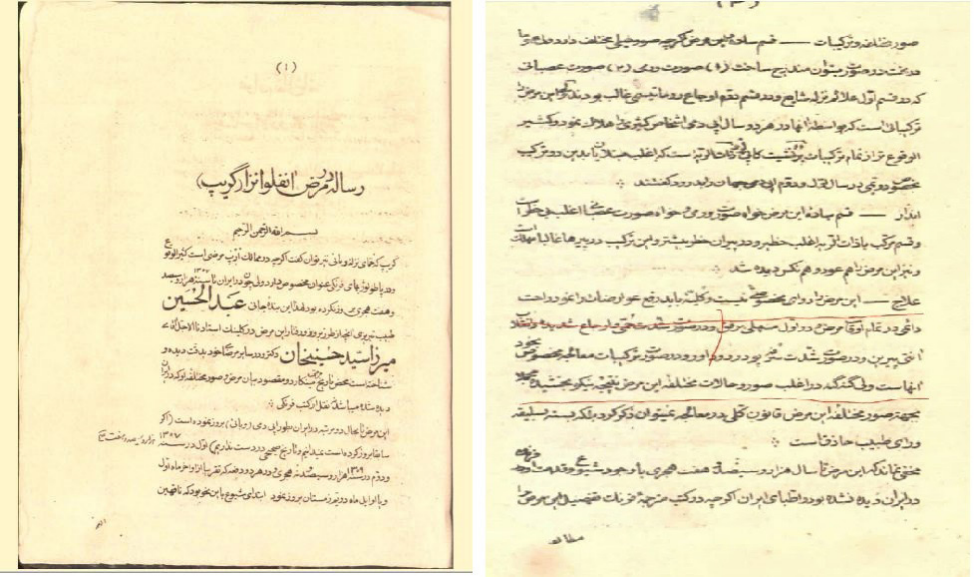
Manuscript Image Grippe or a Treatise on Influenza^32^

 In his “Treatise on Influenza,” Zonuzi documented the influenza outbreak in Tabriz in the late 1890s and early 1891s, which infected about two-thirds of the population.^33^ He provided detailed scientific, pathological, and clinical observations of the disease, noting that it began with slight fatigue, lassitude, lack of appetite, and weakness of the body and muscles. This was followed by fever, cough, runny nose and sneezing, sore throat, earache, and occasionally eye pain, chest pain, and some disability in one hand or foot^32^ (Figure 5).

 The disease caused diarrhea, eclampsia, and miscarriages in pregnant women. Zonuzi identified two forms: vomiting and neurological symptoms, often fatal. He noted that the ancient terms “zokam” and “nazlah” were mistaken for influenza. Avicenna had previously recorded all influenza symptoms, including vomiting, diarrhea, and sore throat.^4,32^ The disease caused body pains and pneumonia, with its origins traced back to Europe. While no specific treatment was widely found, the antimalarial agent quinine showed beneficial effects.^32^

 Zonuzi, as head of Tabriz’s health department, made healthcare accessible to the poor, providing free services and medicine. His efforts likely reduced casualties during the 1918 Spanish flu outbreak in Tabriz, despite half of the population being affected. However, the impact on rural areas remains unknown^13,32^

## Dr. Haidar Mirza Shahrokhshahi

**Figure 6:**
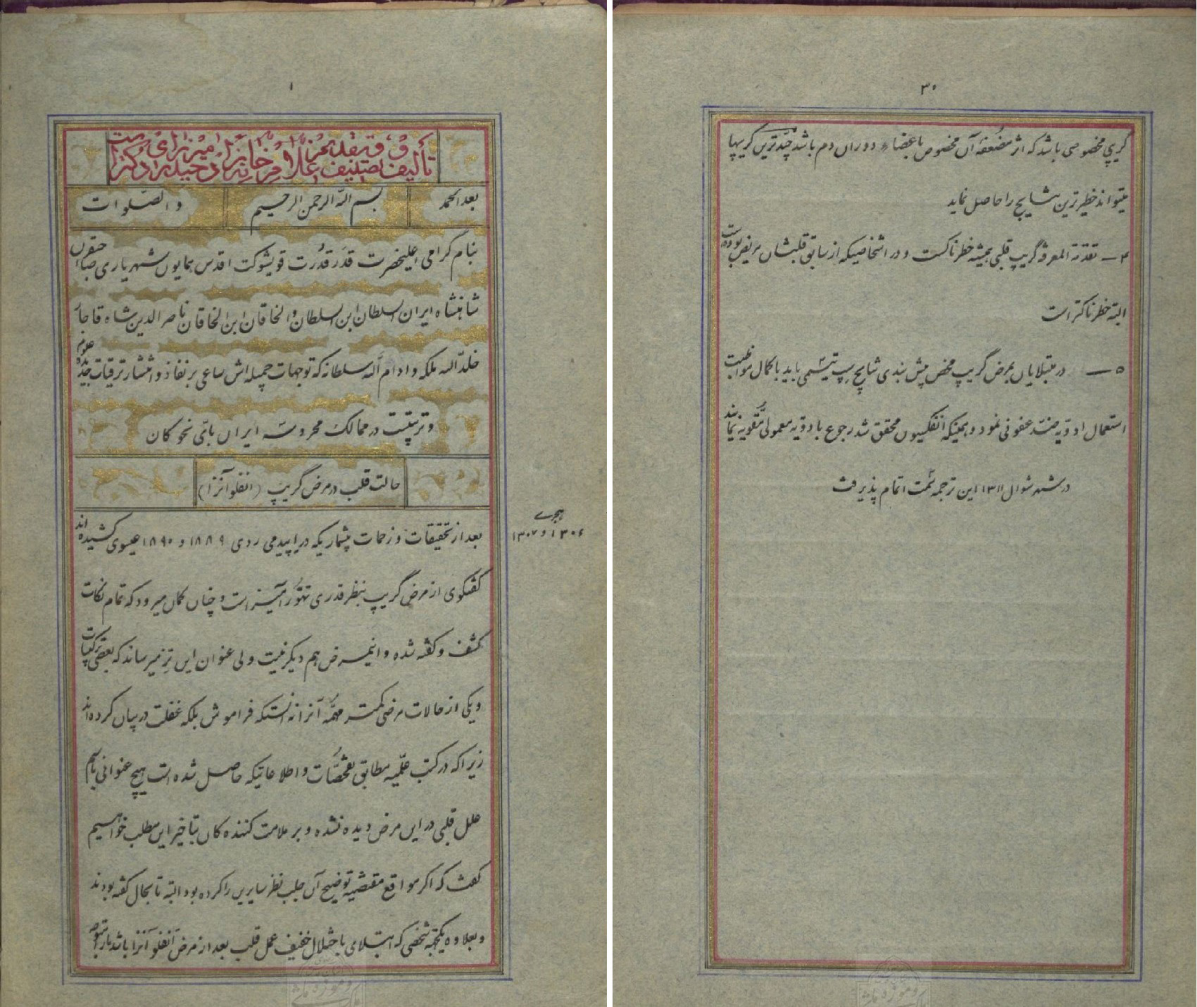
Resaleh dar Maraz Grippe (Influenza)^34^

 Dr. Haidar Mirza Shahrokhshahi, also known as Haidar Mirza-ye Doctor (died 1922), a descendant of the Afshari dynasty (r. 1750-1796) and a notable luminary in Iranian medical history. He was a student of Jule Rishar and Dr. Joseph Désiré Tholozan, receiving his medical diploma under Tholozan’s supervision. In 1894, he wrote “Treatise Grippe,” a thirty-page essay on influenza in honor of Naser al-Din Shah Qajar. This manuscript, influenced by French doctors Mr. Olmon, Mr. Baripe, and Mr. Faisin-Gerre, is archived in the Malek National Library & Museum. It details the 1889-1890 epidemic and offers medical treatments based on Iranian traditional methods^34^ (Figure 6).

 His treatise is divided into five sections, covering the effects of grippe on the heart, the medical rationale for heart disease, disorders of the heart’s function, therapy and early illness recognition, and collected observations highlighting essential facts. The treatise concludes that grippe weakens the heart, thereby increasing the risk of atherosclerosis. It can lead to heart infections, with *Streptococcus pneumoniae* causing septicemia. Furthermore, secondary infections and toxicity from grippe can impair the vascular system, contributing to heart disease. Severe cases of grippe tend to have the most disastrous outcomes. Additionally, heart grippe is especially harmful to people with a history of heart issues.^34^

## Dr. Abul Hasan Khan-e Bahrami (Mirza AbolhassanKkhanSartip)

 Dr. Abul Hasan Khan-e Bahrami, also known as Hasan Khan-e Sartip (1845-1914), was the third Iranian to recognize the significance of the Russian flu and write an essay about it (Essay on grippe). This manuscript is part of Iran’s National Library and Archives collection, though the exact date of its authorship is unknown. Efforts to obtain the text from libraries or archives have been unsuccessful.^13,15,35^

## Conclusion

 The Russian flu, a respiratory disease first identified in 1889 in the Russian Empire, spread globally until 1894, with three peaks, significantly affecting Iran during the second peak. Research papers have inadequately addressed this epidemic in Iran, generally referring to flu. This study draws on historical records and reports from the Istanbul-issued Akhtar magazine. An intriguing aspect of Qajar society was the public interest in microbes and microorganisms responsible for the epidemic. The term “flu” was first used in Persian medical history during this period. Notably, three physicians from Darolfonoon wrote essays on influenza.

 Immunologists’ theories add interest to the historical analysis of the outbreak. They believe that the Russian flu is quite similar to COVID-19, a disease caused by SARS-CoV-2. Many studies have noted parallels between the current COVID-19 pandemic and the Russian flu. For instance, numerous schools and workplaces were shuttered during the Russian flu outbreak, just as they were during the pandemic. Victims of this virus typically lost their senses of taste and smell, while some suffered long-term symptoms lasting months. In general, the Russian flu differed from other flu varieties in that it mostly killed the elderly, although it was deadly to people of all ages. To summarize, the Russian flu was remarkably similar to COVID-19, suggesting that they might share a common origin, though this is still unknown and requires further laboratory testing.
